# The Small RNA CyaR Activates Translation of the Outer Membrane Haem Receptor *chuA* in Enterohemorrhagic *Escherichia coli*

**DOI:** 10.3389/fmicb.2022.821196

**Published:** 2022-03-29

**Authors:** Brandon M. Sy, Jai J. Tree

**Affiliations:** School of Biotechnology and Biomolecular Sciences, University of New South Wales, Sydney, NSW, Australia

**Keywords:** small RNA, Hfq, heme, iron, post-transcriptional, non-coding RNA, EHEC

## Abstract

To sense the transition from environment to host, bacteria use a range of environmental cues to control expression of virulence genes. Iron is tightly sequestered in host tissues and in the human pathogen enterohemorrhagic *Escherichia coli* (EHEC) iron-limitation induces transcription of the outer membrane haem transporter encoded by *chuAS*. ChuA expression is post-transcriptionally activated at 37°C by a FourU RNA thermometer ensuring that the haem receptor is only expressed under low iron, high temperature conditions that indicate the host. Here we demonstrate that expression of *chuA* is also independently regulated by the cAMP-responsive small RNA (sRNA) CyaR and transcriptional terminator Rho. These results indicate that *chuAS* expression is regulated at the transcription initiation, transcript elongation, and translational level. We speculate that additional sensing of the gluconeogenic environment allows further precision in determining when EHEC is at the gastrointestinal epithelium of the host. With previous studies, it appears that the *chuAS* transcript is controlled by eight regulatory inputs that control expression through six different transcriptional and post-transcriptional mechanisms. The results highlight the ability of regulatory sRNAs to integrate multiple environmental signals into a layered hierarchy of signal input.

## Introduction

During infection, nutrients required by pathogenic bacteria are withheld by the host in a process known as nutritional immunity (Weinberg, 1975; Hood and Skaar, 2012). An example can be found in iron, a transition metal that is essential for pathogenic bacteria due to its role in essential physiological processes, such as respiration (Skaar, 2010; Evstatiev and Gasche, 2012). To deprive pathogens of this vital nutrient, the host sequesters iron in the high affinity iron binding molecules haem, ferritin and transferrin (Barber and Elde, 2014; Cornelissen, 2018). These ensure that the concentration of extracellular free iron in the host is too low to support bacterial growth and infection. To overcome this nutritional deficit, pathogens such as *Haemophilus influenzae, Campylobacter jejuni, Pseudomonas aeruginosa*, and uropathogenic *Escherichia coli* have evolved multiple systems that allow for the scavenging of iron, these include siderophores and haem uptake systems (Rossi et al., 2001; Torres et al., 2001; Ong et al., 2006; Ridley et al., 2006; Hagan and Mobley, 2009; Skaar, 2010; Fournier et al., 2011; Garcia et al., 2011; Morgenthau et al., 2013; Zambolin et al., 2016).

Enterohemorrhagic *E. coli* (EHEC) is an enteric pathogen that causes haemorrhagic colitis which can progress into potentially fatal hemolytic uremic syndrome. The primary reservoir of this pathogen are ruminants and transmission mainly occurs *via* the consumption of contaminated foodstuffs (Kaper et al., 2004; Gyles, 2007; Karpman and Ståhl, 2014). EHEC can express an outer membrane haem receptor from the iron regulated *chu* haem uptake operon (Torres and Payne, 1997). The *chu* locus is homologous to the *shu* locus found in *Shigella*, and contains genes that allow for haem uptake, transport, utilization and degradation. The locus consists of a bicistronic operon (*chuAS)* as well as two polycistronic operons (*chuTWXY* and *chuUV*). ChuA is a TonB-dependent outer membrane haem receptor that imports haem into the periplasm (Celia et al., 2016). The periplasmic binding protein ChuT binds to haem, and *via* the ABC transporter ChuUV, is shuttled into the cytoplasm. Haem is processed by the haem oxygenase ChuS that converts haem into biliverdin, carbon monoxide and free iron. This breakdown also prevents haem toxicity (Anzaldi and Skaar, 2010). Under anaerobic conditions, this role is taken up by the SAM-methyltransferase ChuW and the anaerobilin reductase ChuY (LaMattina et al., 2016, 2017).

The haem uptake operon is part of a larger regulatory network that allows EHEC to achieve iron homeostasis. Transcription of *chuA* is repressed by the iron-dependent transcriptional regulator Fur. In iron-rich conditions, Fur binds to iron which increases the affinity of Fur for its DNA-binding site ∼1000-fold (Bags and Neilands, 1987). Fur also regulates a Hfq-dependent small RNA (sRNA) RyhB that is central to maintaining iron homeostasis (Massé and Gottesman, 2002; Massé et al., 2007; Waters and Storz, 2009; Wagner and Romby, 2015). RyhB prevents the expression of non-essential proteins that require Fe as a cofactor such as the TCA cycle and respiration genes *sdhC* and *fumAC*, and iron storage genes such as bacterioferritin. Genes that contribute to iron uptake, such as the shikimate permease *shiA* and the colicin I receptor *cirA*, are positively regulated by this sRNA (Massé and Gottesman, 2002; Oglesby-Sherrouse and Murphy, 2013; Chareyre and Mandin, 2018).

Enterohemorrhagic *Escherichia coli* utilizes the Chu system when it is in an iron-poor, haem-rich host. For that reason, as well as the toxicity that accompanies haem and iron over-accumulation, it is necessary to precisely control expression of this operon. FourU RNA-thermometers are used in different bacterial pathogens, such as *Shigella dysenteriae, Yersinia pseudotuberculosis, Vibrio cholerae*, and *Salmonella typhimurium*, to regulate expression of their virulence factors in a temperature-dependent manner (Waldminghaus et al., 2007; Kouse et al., 2013; Weber et al., 2014; Righetti et al., 2016). A FourU RNA-thermometer was found in the *chuA* 5′UTR that blocks the ribosomal binding site (RBS) and is formed at temperatures below 37°C, indicating that the pathogen is outside of the host (Kouse et al., 2013). This limits *chuA* expression outside of the host where haem is unlikely to be available.

Identification of binding sites for the small RNA chaperone Hfq in EHEC identified the phage-encoded sRNA AsxR that positively regulates the haem oxygenase *chuS* by sponging interactions with the negatively regulating sRNA, FnrS (Tree et al., 2014). Further, analysis of the EHEC sRNA interactome revealed that the outer membrane haem receptor *chuA* was also negatively regulated by the EHEC-specific Hfq-binding sRNA Esr41 (Waters et al., 2017). Here, we show that translation of *chuA* is activated by the Crp-cAMP regulated sRNA CyaR in a temperature- and Rho-termination-independent manner. Our results suggest that EHEC employs an AND-OR logic gate that integrates information on iron availability, temperature and carbon availability to sense its location within the host for appropriate expression of *chuA.*

## Results

### The Outer Membrane Haem Receptor *chuA* Is Regulated by CyaR and ChiX

**FIGURE 1:**
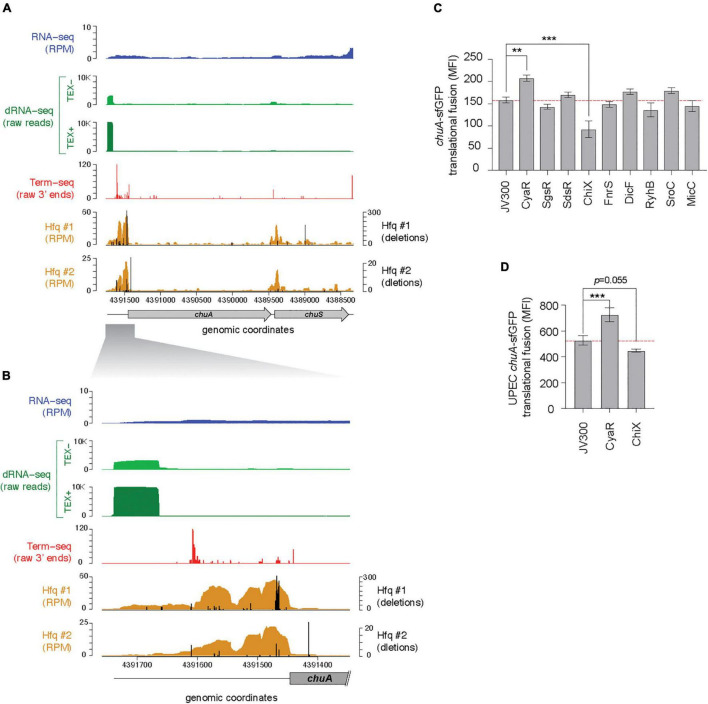
The *chuA* 5′UTR is regulated by Hfq-dependent small RNAs. **(A)** RNA-sequencing data mapping to the *chuAS* operon. From top to bottom: total RNA-seq (dark blue; RPM), differential RNA-seq in samples untreated (light green) and treated (dark green) with TEX, Term-seq (red; raw 3′ end reads) (BioProject PRJNA601151), Hfq-binding sites from UV-crosslinking datasets (BioProject PRJNA197291). Sequencing deletions which indicate direct contact with Hfq are indicated in black. **(B)** Same as **(A)**, but focusing on the 5′UTR of *chuA.* **(C)** Fluorescence measurements of the EHEC *chuA-*sfGFP fusion in the presence of predicted binding sRNAs. **(D)** Fluorescence measurements of the UPEC *chuA-*sfGFP fusion in the presence of CyaR or ChiX (***p < 0.001, **p < 0.01).

In previous work the binding sites of the small RNA chaperone Hfq were mapped using UV-crosslinking, denaturing purification and sequencing of Hfq-crosslinked RNA (termed CRAC) (Tree et al., 2014). Deletions introduced at the site of protein-RNA crosslinking during reverse transcription of UV-crosslinked RNAs can be used to identify the site of direct Hfq-RNA contact. Analysis of our previous Hfq-CRAC dataset reveals that Hfq binds strongly to the 5′UTR of the outer membrane haem receptor *chuA* (Figures 1A,B). Canonically, gene regulation through sRNAs occurs through direct base-pairing of the sRNA to the RBS within the 5′UTR of mRNA targets. Hfq-CRAC sequencing reads and contact-dependent deletions peaked at the RBS consistent with RyhB and Esr41 negative regulation at this site (Waters et al., 2017; Banerjee et al., 2020). A further Hfq-binding peak was located at position +160 to +200 suggesting that *chuA* may be subject to additional regulatory inputs within the 5′UTR (Figures 1A,B).

The *chuA* transcription start site is predicted to be ∼300 nt upstream of the *chuA* start codon (Nagy et al., 2001). To precisely map the transcription start site of *chuA* in EHEC str. Sakai, we analyzed our previously published differential RNA-seq (dRNA-seq) data, which enriches for primary transcripts using terminator exonuclease (TEX) (Sharma et al., 2010; Sy et al., 2020). This showed that the +1 site for *chuA* is at position 4,391,862, and that the 5′UTR is 291 nucleotides long, which concurs with previous predictions (Figure 1B; Nagy et al., 2001).

In previous analyses we found that sRNA-mRNA seed sites are often positioned within Hfq-bound reads recovered by Hfq-CRAC, and that the Hfq binding site can be used to restrict the sequence space for predicting sRNA–mRNA interactions (Tree et al., 2014; Wang et al., 2018). To identify sRNAs that may regulate *chuA*, IntaRNA (Busch et al., 2008; Mann et al., 2017) was used to predict interactions between the *chuA* 5′UTR and 44 known Hfq-dependent sRNAs (Li et al., 2013). Small RNAs that were predicted to interact within Hfq read peaks were retained. Through this analysis, we predicted that the sRNAs CyaR, SgrS, SdsR, ChiX, FnrS, RyhB, DicF, SroC, and MicC may have regulatory effects on *chuA*.

To confirm these predicted interactions the 5′UTR of *chuA*, starting from the +1 site up until the 15th codon of the coding sequence was fused to sfGFP by cloning into the plasmid pXG10SF (Corcoran et al., 2012). The fluorescence of this translational fusion was monitored in *E. coli* strain DH5α in the presence and absence of the candidate sRNAs expressed from constitutive expression plasmids (Figure 1C). This revealed that the Hfq-dependent sRNAs CyaR and ChiX have an activating and repressive effect on *chuA*, respectively.

### CyaR Regulation of *chuA* Is Conserved in Uropathogenic *Escherichia coli*

Earlier work from the Murphy Lab indicated that *chuA* 5′UTR from EHEC and *Shigella* are more closely related than the 5′UTR from EHEC and uropathogenic *E. coli* (UPEC) (Kouse et al., 2013). We next examined whether CyaR and ChiX regulation are conserved in the divergent EHEC and UPEC 5′UTRs. In UPEC, haem acquisition using *chuA* is required for maximal colonization of the kidneys (Hagan and Mobley, 2009). The 5′UTRs of EHEC and UPEC (*chuA*_*EHEC*_ and *chuA*_*UPEC*_) *chuA* share 83.4% identity (Nagy et al., 2001). The UPEC 5′UTR sequence has only 67.4% identity within the Hfq-binding site suggesting that *chuA*_*UPEC*_ may not be regulated by Hfq-dependent sRNAs. To test whether *chuA*_*UPEC*_ is post-transcriptionally regulated by CyaR and ChiX we constructed a translational fusion of *chuA*_*UPEC*_ with sfGFP and monitored fluorescence in the presence or absence of CyaR and ChiX overexpression plasmids. The *chuA*_*UPEC*_ fusion was activated by CyaR consistent with our results for *chuA*_*EHEC*_ but was not significantly repressed by ChiX (Figure 1D). Notably, the *chuA*_*UPEC*_ fusion was 55% more fluorescent than the *chuA*_*EHEC*_ fusion. This suggests that while *chuA* in both pathotypes is subject to regulation by CyaR, the divergent 5′UTR sequence has de-repressed *chuA* expression in UPEC.

### CyaR Activates *chuA* Translation Through Direct Base-Pairing

Both CyaR and ChiX are class II sRNAs that are known to modulate sRNA regulation by displacing sRNAs from Hfq when overexpressed (Moon and Gottesman, 2011; Santiago-Frangos et al., 2016). To determine whether CyaR and ChiX control *chuA* through direct RNA–RNA interactions with the *chuA* 5′UTR or indirectly through titration of Hfq, point mutations were made in both *chuA* and the sRNAs CyaR and ChiX to disrupt the predicted interaction sites. A predicted 3 nt compensatory mutation was made in the top scoring *chuA*–ChiX interaction as predicted by IntaRNA. The M1 mutation made in ChiX resulted in the de-repression of *chuA-*GFP expression. However, this effect was not observed when the compensatory *chuA* M1 mutation was introduced. This suggested that while the nucleotides mutated in ChiX contributed to *chuA* regulation, they did not interact with the predicted *chuA* interaction site. A second set of compensatory point mutants (M2) were made for the next highest scoring interaction that used the same ChiX seed region that was previously tested. Mutations in either *chuA* or ChiX alone resulted in the disruption of the *chuA*–ChiX interaction, but testing the mutants together did not restore the repression of *chuA* (Supplementary Figure 1). These results indicated that while ChiX affects translation of *chuA*, it is not due to a direct interaction with the *chuA* M1 or M2 site and may occur indirectly.

**FIGURE 2:**
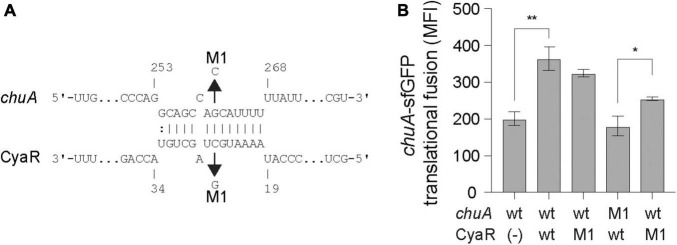
CyaR activates *chuA* translation through direct base-pairing. **(A)** IntaRNA prediction of the *chuA*–CyaR interaction. Compensatory point mutations predicted to disrupt the interaction are indicated by the arrows. **(B)** Fluorescence measurements of wild-type or mutant *chuA-*sfGFP translational fusions in the presence and absence of wild-type or mutant CyaR overexpression plasmid. Measurements are the mean median fluorescence intensity of three biological replicates (**p < 0.01, *p < 0.05).

CyaR is predicted to bind approximately 15 nt upstream of the *chuA* Shine–Dalgarno sequence. The interaction between CyaR and *chuA* results in a 1.3-fold activation of translation. Mutating CyaR at the M1 site reduced CyaR activation by a modest 8.1%, while the *chuA* point mutant completely abolished the interaction (Figure 2). Providing the CyaR-M1 mutant partially restore translational activation of *chuA*-M1, supporting a direct interaction between CyaR and the 5′UTR of *chuA* that activates translation.

### CyaR Activates ChuA Translation Independently of Temperature

**FIGURE 3:**
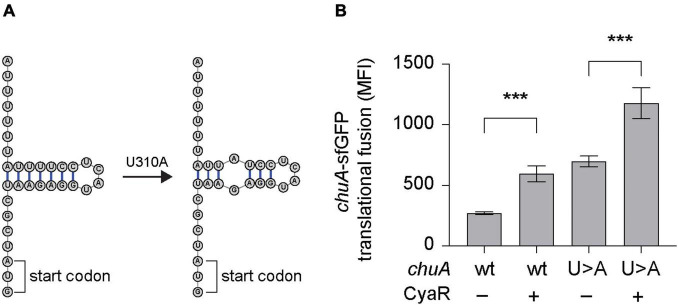
CyaR activates *chuA* translation independently of a FourU RNA-thermometer. **(A)** Diagram indicating the point mutation made to disrupt the formation of the *chuA* FourU RNA thermometer. **(B)** Fluorescence measurements of wild-type and RNA-thermometer disrupted *chuA-*sfGFP translational fusions in the presence and absence of CyaR overexpression plasmid. Measurements are the mean median fluorescence intensity of three biological replicates (***p < 0.001).

Regulation of *chuA* occurs on both the transcriptional and translational level. Transcription of *chuA* is inhibited by Fur when cells are grown in iron-rich conditions (Torres and Payne, 1997), while the level of translation is dependent on the environmental temperature. At 25°C, translation is inhibited by the formation of a FourU RNA thermometer that occludes the RBS (Kouse et al., 2013). This inhibitory structure lies 15 nt downstream of the CyaR binding site. To determine whether CyaR-mediated activation of *chuA* translation occurs by inhibiting the formation of this RNA-thermometer, a U273A point mutation known to disrupt the formation of the FourU hairpin loop was made in the 5′UTR of the *chuA-*GFP translational fusion (Figure 3A; Kouse et al., 2013). Expression of wild-type and mutant *chuA*-sfGFP translational fusions were monitored in the presence and absence of CyaR overexpression plasmids at 25°C. Disruption of the RNA thermometer *via* the U273A point mutation resulted in a 2.6-fold increase in fluorescence compared to the wild-type, confirming the FourU temperature-dependent regulation of *chuA* translation. Disruption of this secondary structure however did not affect CyaR-mediated activation of *chuA*, as a 1.6-fold increase in fluorescence was observed when CyaR was provided with the U273A mutation in *chuA* (Figure 3B). This demonstrated that CyaR-mediated activation of *chuA* acts independently of post-transcriptional inhibition through the FourU RNA thermometer structure.

### The *chuA* 5′UTR Contains a Sequence That Inhibits Its Expression

**FIGURE 4:**
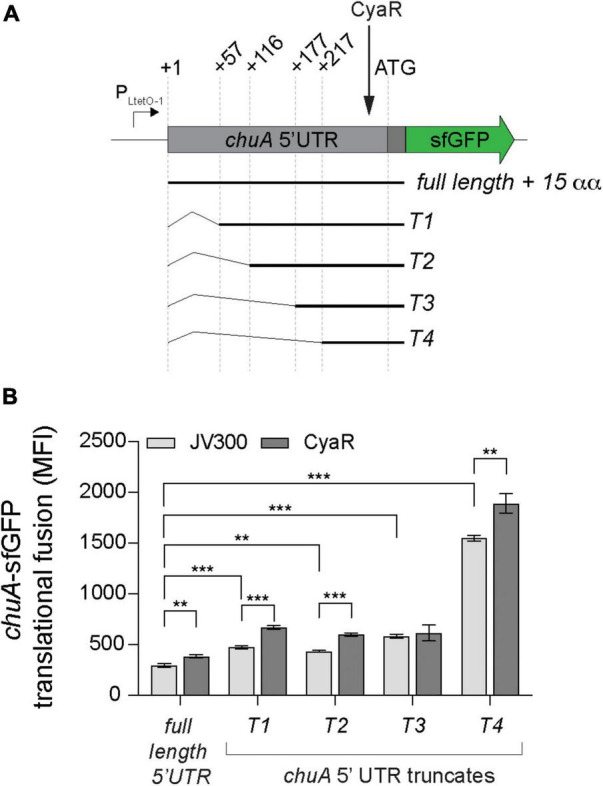
The *chuA* 5′UTR contains a sequence that disrupts its translation independently of CyaR. **(A)** Diagram indicating the positions of the different *chuA-*sfGFP truncates used. **(B)** Fluorescence measurements of full-length and truncated *chuA-*sfGFP constructs in the presence and absence of CyaR overexpression plasmid. The position of the CyaR binding site is indicated. Measurements are the mean median fluorescence intensity of three biological replicates (***p < 0.001, **p < 0.01).

While the activation of *chuA* translation by CyaR is not due to disruption of the RNA thermometer, *in silico* folding predictions on the 5′UTR of *chuA* indicated potential secondary structure throughout the 5′UTR, including some that occluded sequences downstream of the RBS (Supplementary Figure 2). To understand whether the activating *chuA*–CyaR interaction requires structured sequences within the *chuA* 5′UTR, we progressively removed sequence from the 5’ end in four truncations of the *chuA-*sfGFP translational fusion (Figure 4A). Predicted secondary structure and the presence of Hfq distal face binding motifs [(ARN)_*x*_] were used as guides in making truncates of the *chuA* 5′UTR (Soper and Woodson, 2008; Zhang et al., 2013; Schu et al., 2015). A search through the 5′UTR of *chuA* identified 10 (ARN)_4_ sites with one mismatch tolerated (ARN_4_m_1_). The first truncate (T1) begins at +57 nt and removes a section that forms two hairpins as well as 9 out of 10 ARN_4_m_1_ sites. The second (T2) and third (T3) begin at +116 and +177, respectively, and each one removes another hairpin from the overall predicted secondary structure while maintaining the RNA-thermometer. The second truncate T2 also removes the last ARN_4_m_1_ motif. The fourth truncate (T4) begins at +217 and leaves only the CyaR binding site, the RNA-thermometer, RBS and the start codon. In the absence of the CyaR overexpression plasmid, the *chuA* 5′UTR T1-T4 truncates caused 2.1-, 1.9-, 2.9-, and 9.2-fold increases in *chuA* translation, respectively. CyaR was able to activate truncates T1, T2, and T4 between 20 and 40% consistent with CyaR activation of the wild-type construct (Figure 4B). These results indicate that CyaR does not act through alleviation of an inhibitory secondary structure or a regulatory sequence in the upstream +1 to +217 nt region of the 5′UTR. Notably, removing the region between +177 and +217 nt (T3–T4) of the *chuA* 5′UTR resulted in a dramatic increase in *chuA-*GFP expression (9.2-fold) indicating that the T3–T4 region of the 5′UTR contains sequences or RNA structures that strongly repress expression.

### *chuA* Is Subject to Premature Transcription Termination by Rho

**FIGURE 5:**
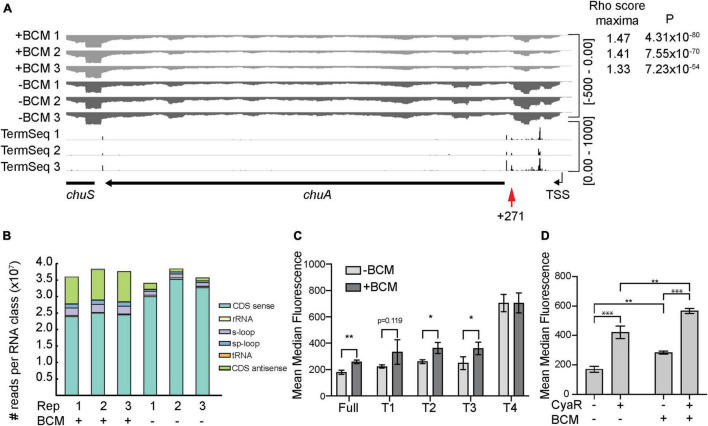
The *chuA* 5′UTR is subject to termination by Rho. **(A)** RNA-seq coverage of *chuA* in EHEC O157:H7 strain Sakai treated and untreated with bicyclomycin. Rho scores for each replicate is shown. TSS is derived from differential RNA-seq data and is indicated by an arrow. Term-seq reads indicating *chuA* 3′ ends are also included. **(B)** Classification and number of reads per RNA class upon treatment of EHEC O157:H7 strain Sakai with bicyclomycin. **(C)** Fluorescence measurements of full-length and truncated *chuA-*sfGFP fusions in the presence and absence of induction with bicyclomycin. **(D)** Fluorescence measurements of *chuA-*sfGFP translational fusion in the presence and absence of CyaR overexpression plasmid and treatment with bicyclomycin. Measurements are the mean median fluorescence intensity of three biological replicates (***p < 0.001, **p < 0.01, *p < 0.05).

In *E. coli*, the average 5′UTR is approximately 25–35 nucleotides in length (Kim et al., 2012; Evfratov et al., 2017). The 5′UTR of *chuA* is 291 nucleotides, making it an unusually long UTR (Wyckoff et al., 1998; Kouse et al., 2013). In commensal *E. coli*, over half of all annotated genes with long 5′UTRs (defined as >80 nt) are prematurely terminated by the transcription termination factor Rho (Sedlyarova et al., 2016). Further, horizontally transferred genes have been shown to be more susceptible to regulation by Rho-dependent termination (Cardinale et al., 2008; Peters et al., 2012). By analyzing our previously published Term-seq data, we noticed that there were termination sites at positions +129, +271, and +297 within the *chuA* mRNA (Figure 5A). We did not identify Rho-independent (intrinsic) terminators within the *chuA* 5′UTR using ARNold (Figure 5A; Naville et al., 2011; Sy et al., 2020). Collectively, this suggested that transcription of the pathogenicity island encoded *chuA* may be prematurely terminated by Rho.

To identify Rho-termination sites in EHEC, we sequenced total RNA extracted from cells treated with the Rho-inhibiting antibiotic bicyclomycin (BCM) (Zwiefka et al., 1993). Rho-termination sites were identified by using the approach adopted by Adams et al. (2021) that previously identified termination sites in BCM-treated *E. coli* cells. Consistent with earlier studies we found that horizontally acquired regions and antisense RNAs are enriched among genes that are up-regulated by BCM treatment (Figure 5B; Cardinale et al., 2008; Peters et al., 2009). Read-through scores were calculated to assess Rho-dependent termination using previously published scripts (Adams et al., 2021). Read-through scores were maximal for all three replicates (±BCM) at positions +267–273 nt correlating well with the termination site at +271 nt identified by Term-seq. Read-through transcription at this site was up-regulated between 1.33- and 1.47-fold by BCM treatment indicating that Rho prematurely terminates transcription of *chuA* at position +271 (Figure 5A). To verify that *chuA* was subject to premature transcription termination by Rho, we measured the fluorescence of inducible full-length and truncated *chuA-*GFP translational fusions in the presence or absence of BCM (Figure 5A). Treatment with BCM resulted in a ∼1.4-fold increase in fluorescence for the full-length and T1–T3 *chuA-*GFP constructs. No significant change was observed for *chuA* truncate T4 (Figure 5C). Rho-dependent termination requires recruitment of the Rho at upstream Rho utilization sites (*rut*) and we speculate that the *rut* site for Rho-dependent termination at +271 is removed in the T4 truncate. Taken together, these results indicate that *chuA* is negatively regulated by Rho-dependent termination at position +271 and this repression requires the 30 nt between +177 and +217 nt of the *chuA* 5′UTR.

Small RNAs can regulate Rho-dependent termination by altering the accessibility of Rho utilization sites, either as a by-product of translation inhibition, or by directly binding to the *rut* site itself (Bossi et al., 2012; Sedlyarova et al., 2016; Chen et al., 2019; Silva et al., 2019). We hypothesized that CyaR may activate *chuA* translation by preventing premature Rho termination within the 5′UTR. To test this, we measured the fluorescence of our *chuA-*GFP translational fusion in the presence or absence of BCM and CyaR. CyaR was able to activate translation of *chuA* in the presence of BCM, indicating that CyaR activates *chuA* independently of Rho termination (Figure 5D). Collectively our data demonstrate that *chuA* expression is independently regulated by CyaR sRNA, Rho terminator, and the FourU RNA thermometer.

### Expression of CyaR Is Correlated With *chuA* Expression *in vivo*

**FIGURE 6:**
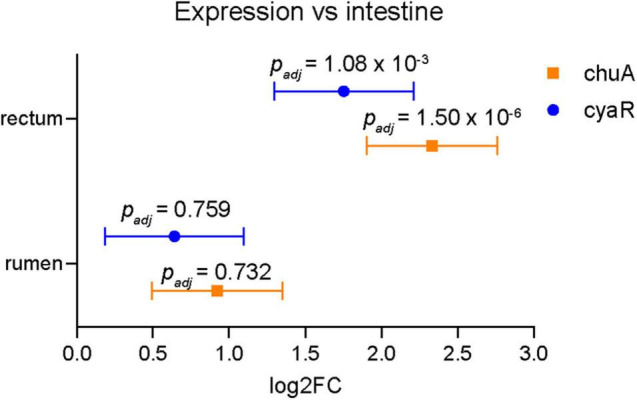
Expression of chuA and CyaR in bovine gastrointestinal niches. Log2FC expression of chuA (orange squares) and CyaR (blue circles) in rectum and rumen compared to intestine. Data from SRA accession SRP136076. Error values are standard errors calculated from DEseq2. Adjusted *p*-values are calculated from DEseq2.

In order to obtain a more biologically relevant representation of the relationship between CyaR and *chuA* expression, we analyzed previously published RNA sequencing data of EHEC str. EDL933 obtained during colonization of a bovine rectum, intestine and rumen (Segura et al., 2018, 2021). Previously, these datasets have been used to show that genes relating to iron acquisition are differentially regulated in each of the gastrointestinal niches when compared to minimal M9 media. We compared the expression of both *chuA* and CyaR between each of the gastrointestinal niches and we find that *chuA* is most highly expressed in the rectum, followed by the rumen, then the intestine (Figure 6). These findings are consistent with induction of *chuA* by environmental signals at this site, including iron-limitation, temperature and a gluconeogenic environment (Snider et al., 2009; Bertin et al., 2013). We also observe this trend with CyaR expression, with the sRNA being maximally expressed in the rectum. This suggests that CyaR contributes to the maximal expression of *chuA* in the rectum, allowing for better colonization of this environmental niche.

## Discussion

To successfully grow and colonize a host, pathogens utilize systems that allow them to retrieve trace minerals required for growth that are normally sequestered. Various pathotypes of *E. coli*, including EHEC and UPEC, utilize the ChuA outer membrane haem receptor to transport host derived haem. Transcription of *chuA* is repressed in the presence of iron by Fur (Torres and Payne, 1997), while translation is regulated by temperature through a FourU RNA-thermometer that occludes the RBS (Kouse et al., 2013). The combination of these two modes of regulation allows the pathogen to sense two signals associated with the host environment (low iron and high temperature) and activate expression of the haem receptor inside the host. Notably, each signal in isolation would generate many false positives when deciding on whether the cell had entered a mammalian host.

**FIGURE 7:**
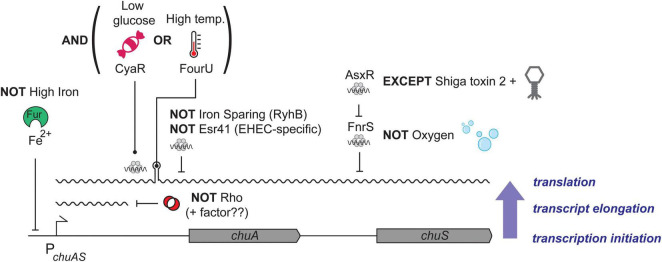
Model of *chuAS* regulation. Regulation of *chuAS* expression occurs at the transcriptional, transcript elongation, and translational levels (indicated blue, right). The dependence of each layer on the previous creates regulatory AND gates. (*Bottom layer*) Repression of *chuAS* transcription by Fe-loaded Fur is relieved under iron-limited conditions allowing transcription initiation. (*Middle layer*) Elongating RNAP is prematurely terminated by Rho within the 5′UTR of *chuA*. An additional factor (potentially another sRNA) is likely to modulate Rho termination and allow read-through transcription. (*Top layer*) Translation of the *chuAS* transcript is activated by the cAMP-responsive sRNA CyaR or temperatures ł37°C that melt the FourU thermometer. Melting of the *chuAS* mRNA FourU element can be overridden under iron-sparing conditions by the sRNA RyhB or the EHEC-specific sRNA Esr41 that repress translation from the exposed RBS. The downstream haem oxygenase encoded by *chuS* is repressed under aerobic conditions by the sRNA FnrS, except in EHEC where repression is relieved by the sRNA sponge AsxR carried by the Shiga toxin 2-encoding bacteriophage.

The natural order imposed by transcription and post-transcriptional regulation creates an AND-logic gate, where low iron levels AND high temperature are required for expression of *chuA* in the host. Here we have shown that *chuA* is controlled by two additional post-transcriptional signals: repression through Rho termination and activation through CyaR. CyaR acts independently of both Rho and the FourU thermometer potentially creating a post-transcriptional OR-logic gate. The effect of the FourU thermometer and CyaR are additive, and either regulator can activate independently of the other. In Boolean terms it appears that *chuA* uses an AND-OR logic gate where expression requires low iron AND (high temperature OR CyaR) (Figure 7).

Transcription of CyaR is activated by the global regulator Crp when cyclic-AMP levels are high, such as in the absence of glucose (De Lay and Gottesman, 2009). EHEC colonizes the colon where the primary source of carbon is mucins. *Bacteroides thetaiotaomicron (Bt)* can cleave mucins to release sugars that are utilized by EHEC (Xu et al., 2003; Fischbach and Sonnenburg, 2011). During colonization of the gastrointestinal mucus layer and epithelium, EHEC is likely to encounter varying niches that include both glycolytic and gluconeogenic environments (Miranda et al., 2004). Sensing variation in the availability of sugars and oxygen availability have been suggested to be key regulatory signals that allow EHEC to determine location in the gastrointestinal tract and proximity to the epithelium (Carlson-Banning and Sperandio, 2016). Close to the intestinal epithelium, the relative absence of microorganisms that metabolize and release sugars from mucins creates at gluconeogenic environment (Conway and Cohen, 2015). Lower levels of fucose, and higher levels of succinate reduces the level of the fucose-sensing two-component system FusKR that induces expression of Cra. In gluconeogenic environments, Cra can enhance binding of the cAMP receptor protein Crp to its targets (Ryu et al., 1995). Activation of Cra can induce expression of genes required for T3SS and adhesion to the epithelium (Njoroge et al., 2012; Curtis et al., 2014; Carlson-Banning and Sperandio, 2016). Expression of Crp can also be activated in the presence of Cra (Zhang et al., 2014). We propose that the gluconeogenic environment encountered at the gastrointestinal tract provides an activating signal for CyaR that has been incorporated into *chuA* regulation as an additional signal that indicates host colonization and haem availability. Consistent with this hypothesis, we find that both CyaR and *chuA* transcription is activated in bovine rectal samples (Figure 6).

The mechanism of CyaR activation of *chuA* expression remains unclear although we have ruled out three probable mechanisms: CyaR acts through direct base-pairing with the *chuA* 5′UTR rather than general titration of Hfq, CyaR does not disrupt the FourU RNA thermometer to override temperature-dependence, and CyaR does not inhibit premature Rho termination of *chuA*. While these results have uncovered two additional post-transcriptional regulators of *chuA* (CyaR and Rho) they also suggest that there exists an additional repressive element in the *chuA* 5′UTR that is overcome by CyaR binding. We note that our *chuA-*GFP translational fusion reports on multiple post-transcriptional processes including translation efficiency and mRNA stability, and that CyaR may be modulating either of these to affect *chuA* expression.

Our Term-seq data indicates that the *chuA* 5′UTR is terminated at positions +129, +271, and +297 with position +271 nt maximally sensitive to treatment with BCM. Using truncates of the *chuA* 5′UTR we have also found that the sequence between +177 and +217 nt (T3 and T4) represses *chuA* expression almost 10-fold. We suggest that Rho associates with the nascent *chuA* transcript between +177 and +217 nt to terminate transcription at +271 nt. Small RNAs can promote or occlude *rut* sites to control Rho termination in response to regulatory signals (Sedlyarova et al., 2016; Silva et al., 2019) and it remains plausible that Rho termination is modulated by yet another regulatory signal.

The *chuA* encoded haem receptor is transcribed as a bicistronic transcript with the haem oxygenase *chuS*. In previous work we showed that *chuS* expression is repressed by the sRNA FnrS that is induced under anaerobic conditions, consistent with ChuS requiring oxygen for activity. FnrS repression is over-ridden by the sRNA sponge AsxR transcribed from the Shiga toxin 2 encoding bacteriophage Sp5 (Tree et al., 2014). Collectively, expression of *chuAS* is subject to an impressive level of post-transcriptional regulation that provides complex integration of environmental signals beyond low iron (transcriptional regulation by Fur). These post-transcriptional signals appear to provide a much more accurate interpretation of the environment to indicate whether haem transport is required (i.e., whether EHEC is at the gastrointestinal epithelium). The logic of the regulatory circuit that controls *chuAS* expression is outlined in Figure 7.

Post-transcriptional regulation appears ideally suited to this layering of logic gates because the natural order (dependence) imposed by transcriptional and post-transcriptional signals creates AND gates. There are at least three ordered layers of AND gates in the *chuA* transcript that need to be satisfied before the next layer of signals are incorporated. Fur-dependent transcription, Rho-dependent termination, and post-transcriptional control through sRNAs or the FourU thermometer. Together these form an elegant set of AND, OR, and NOT gates that interpret the environment and genetic background of the host (the later through the EHEC-specific sRNAs AsxR and Esr41) (Figure 7). The extensive post-transcriptional logic of the *chuAS* operon suggests that layered post-transcriptional regulation can create complex regulatory logic gates to integrate and interpret environmental signals.

## Materials and Methods

### Bacterial Strains and Growth Conditions

Bacterial strains, oligonucleotides and plasmids used for this study are listed in Supplementary Table 1. *E. coli* was routinely grown at 37°C in liquid Luria-Bertani (LB) broth or on solid LB-agar plates. Bacterial media was supplemented with ampicillin (100 μg/mL) or chloramphenicol (34 μg/mL) where appropriate. For inhibition of Rho termination, bicyclomycin (BioAustralis, 50 μg/mL) was added to exponential phase (OD_600_ = 0.6) cultures for 30 min before being assayed.

### *In silico* Prediction of Interacting sRNAs

A list of published sRNAs present in enterohemorrhagic *E. coli* was taken from the Bacterial Small Regulatory RNA Database (Li et al., 2013). sRNAs listed as not being Hfq-binding were filtered out, and the sequences of the remaining sRNAs were input into IntaRNA (Busch et al., 2008; Wright et al., 2014; Mann et al., 2017) to search for interactions with the 5′UTR of *chuA*. sRNAs that were predicted to bind to regions of the *chuA* 5′UTR that were not Hfq-binding were removed from consideration.

### Construction of GFP-Translational Fusions and sRNA Expression Vectors for Testing sRNA–mRNA Interactions

Plasmids pXG10SF containing the full-length and truncated *chuA-*GFP translational fusion and pZE12 carrying candidate sRNAs were cloned according to method described in Urban and Vogel (2007). Briefly, the 5′UTR of *chuA* and candidate sRNAs were amplified from genomic DNA using primers identified in Supplementary Table 1. The *chuA-*GFP translational fusion was inserted using *Nhe*I-*Nsi*I restriction cloning. Candidate sRNAs were inserted by first linearizing pZE12-*luc* using primers pL*lac*_*O*_-B and pL*lac*_*O*_-D. The linearized amplicon, as well as the candidate sRNA amplicon were digested using *Xba*I and ligated together using T4 DNA ligase.

Mutations were made in the *chuA* or sRNA sequences by using the Quikchange XL mutagenesis kit (Agilent) according to the manufacturer’s instructions. Primers for mutagenesis were designed using the Quikchange primer design program.^1^

### Confirmation of sRNA–*chuA* Interactions Using the sfGFP 2-Plasmid System

The expression of sfGFP was monitored and quantified with and without candidate sRNAs using a BD FACSCanto II or a BD LSRFortessa™ Special Order Research Product cell analyzer. Fluorescence was measured using a 530/30 nm bandpass filter. Forward scatter (FSC) and side scatter (SSC) were also measured to gate the bacterial population. For each sample, at least 100,000 events were recorded. Data was analyzed using FlowJo software (BD) and statistics were calculated using Prism 8 (GraphPad) to obtain each sample’s mean median fluorescence intensity (mean MFI). *p*-Values were calculated using a standard two-tailed Student’s *t*-test.

Plasmids expressing the wild-type or mutant *chuA-*GFP translational fusion and those expressing candidate sRNAs were co-transformed into *E. coli* DH5α or TOP10F′. Three colonies from each transformation were purified and grown overnight in LB broth. Overnight cultures are subcultured 1/100 in 0.22 μm filtered LB broth and grown to exponential phase (OD_600_ = 0.6). Expression of the *chuA-*GFP translational fusions were induced in TOP10F′ using 200 nM of anhydrotetracycline. These are diluted fivefold in 0.22 μm-filtered PBS, then read on the flow cytometer as described above. Plasmid pJV300 expressing a scrambled sRNA, and pXG1 and pXG0 plasmids expressing GFP and Lux, respectively, were used as controls.

### RNA Secondary Structure Prediction

The secondary structure for the 5′UTR of *chuA* was predicted using the mfold^2^ and RNAfold webservers (Zuker, 2003; Lorenz et al., 2011). Figures were drawn on RNAStructure version 6.0.1 (Reuter and Mathews, 2010). ARN motifs were detected using custom scripts previously used in Tree et al. (2014).

### RNA Sequencing of Bicyclomycin-Treated Cells

Three single colonies of *E. coli* O157:H7 str. Sakai *stx-* were grown for 16 h in LB broth. The following day, these subcultured 1/100 in MEM-HEPES supplemented with 0.1% glucose and 250 nM Fe(NO_3_)_3_. At an OD_600_ of 0.75, cultures were split into two (treated and untreated), and 50 μg/mL of bicyclomycin was added to the treated sample. Cultures were incubated for a further 15 min, followed by addition of RNA stop solution (5% water-saturated phenol in ethanol). RNA was extracted using guanidinium thiocyanate-phenol as previously described in Tollervey and Mattaj (1987). Genomic DNA was digested using RQ1 DNase (Promega) and RNA was cleaned using another phenol-chloroform extraction. Total RNA was ribodepleted using the Zymo-Seq RiboFree Total RNA library kit and libraries were prepared using the Illumina NextSeq 500/550 Mid-Output Kit. Samples were 2 × 75 bp paired-end sequenced on an Illumina NextSeq 500 platform. Library preparation and sequencing were performed by the Ramaciotti Centre for Genomics in the University of New South Wales, Sydney, NSW, Australia.

### RNA Sequencing Analysis and Identification of Rho-Dependent Transcripts

RNA sequencing data for *in vivo* analysis was taken from SRA accession number SRP136076. Alignment and DEseq analysis of RNA sequencing output was done using the “align,” “coverage,” “gene-quanti,” and “deseq” modules of READemption v1.0 (Förstner et al., 2014) using default settings.

To identify Rho-terminated transcripts, fastq files were re-aligned to the *E. coli* O157:H7 str. Sakai genome (accession number: NC_002695.2) using BWA-MEM (v0.7.17) (Li and Durbin, 2010). BAM files were generated using samtools (v.1.10) (Li et al., 2009) and 3′ end read counts were calculated using the genomecov tool from bedtools (v2.27.1) (Quinlan and Hall, 2010). Rho readthrough scores were calculate using the program RhoTerm-Peaks using a window size of 250 nt (Adams et al., 2021).

## Data Availability Statement

RNA sequencing datasets are deposited at the NCBI Gene Expression Omnibus (GEO) under accession numbers GSE143631, GSE46118, and GSE197379.

## Author Contributions

BS and JT conceived and designed the experimental work, analyzed the data, and wrote the manuscript. BS performed the experimental work. Both authors contributed to the article and approved the submitted version.

## Conflict of Interest

The authors declare that the research was conducted in the absence of any commercial or financial relationships that could be construed as a potential conflict of interest.

## Publisher’s Note

All claims expressed in this article are solely those of the authors and do not necessarily represent those of their affiliated organizations, or those of the publisher, the editors and the reviewers. Any product that may be evaluated in this article, or claim that may be made by its manufacturer, is not guaranteed or endorsed by the publisher.

## Footnotes

1
https://www.agilent.com/store/primerDesignProgram.jsp

2
https://unafold.rna.albany.edu/?q=mfold

## Supplementary Material

Supplementary Figure 1 ChiX indirectly represses *chuA* translation. **(A,B)** (*Left*) IntaRNA prediction of the *chuA*–ChiX interaction. Compensatory point mutations predicted to disrupt the interaction are indicated by the arrows. (*Right*) Fluorescence measurements of wild-type or mutant *chuA-*sfGFP translational fusions in the presence and absence of wild-type or mutant ChiX overexpression plasmid. Measurements are the mean median fluorescence intensity of three biological replicates.

Supplementary Figure 2 Secondary structure of the *chuA* 5′UTR as predicted by RNAfold. The +1 site, CyaR binding site, FourU RNA thermometer and sites where truncations were made are indicated.

Supplementary Table 1 Primers, bacterial strains, and plasmids used in this work.

## References

[B1] Adams P. P. Baniulyte G. Esnault C. Chegireddy K. Singh N. Monge M. (2021). Regulatory roles of *Escherichia coli* 5’ UTR and ORF-internal RNAs detected by 3’ end mapping. *eLife* 10:e62438. 10.7554/eLife.62438 33460557 PMC7815308

[B2] Anzaldi L. L. Skaar E. P. (2010). Overcoming the heme paradox: Heme toxicity and tolerance in bacterial pathogens. *Infect. Immun.* 78 4977–4989. 10.1128/IAI.00613-10 20679437 PMC2981329

[B3] Bags A. Neilands J. B. (1987). Ferric uptake regulation protein acts as a repressor, employing iron(II) as a cofactor to bind the operator of an iron transport operon in *Escherichia coli*. *Biochemistry* 26 5471–5477. 10.1021/bi00391a039 2823881

[B4] Banerjee R. Weisenhorn E. Schwartz K. J. Myers K. S. Glasner J. D. Perna N. T. (2020). Tailoring a global iron regulon to a uropathogen. *mBio* 11:e00351-20. 10.1128/mBio.00351-20 32209682 PMC7157518

[B5] Barber M. F. Elde N. C. (2014). Escape from bacterial iron piracy through rapid evolution of transferrin. *Science* 346 1362–1366. 10.1126/science.1259329 25504720 PMC4455941

[B6] Bertin Y. Chaucheyras-Durand F. Robbe-Masselot C. Durand A. de la Foye A. Harel J. (2013). Carbohydrate utilization by enterohaemorrhagic *Escherichia coli* O157: H7 in bovine intestinal content. *Environ. Microbiol.* 15 610–622. 10.1111/1462-2920.12019 23126484 PMC3558604

[B7] Bossi L. Schwartz A. Guillemardet B. Boudvillain M. Figueroa-Bossi N. (2012). A role for Rho-dependent polarity in gene regulation by a noncoding small RNA. *Genes Dev.* 26 1864–1873. 10.1101/gad.195412.112 22895254 PMC3426764

[B8] Busch A. Richter A. S. Backofen R. (2008). IntaRNA: efficient prediction of bacterial sRNA targets incorporating target site accessibility and seed regions. *Bioinformatics* 24 2849–2856. 10.1093/bioinformatics/btn544 18940824 PMC2639303

[B9] Cardinale C. J. Washburn R. S. Tadigotla V. R. Brown L. M. Gottesman M. E. Nudler E. (2008). Termination factor Rho and its cofactors NusA and NusG silence foreign DNA in *E. coli*. *Science* 320 935–938. 10.1126/science.1152763 18487194 PMC4059013

[B10] Carlson-Banning K. M. Sperandio V. (2016). Catabolite and oxygen regulation of enterohemorrhagic *Escherichia coli* virulence. *mBio* 7:e01852-16. 10.1128/mBio.01852-16 27879335 PMC5120142

[B11] Celia H. Noinaj N. Zakharov S. D. Bordignon E. Botos I. Santamaria M. (2016). Structural insight into the role of the Ton complex in energy transduction. *Nature* 538 60–65. 10.1038/nature19757 27654919 PMC5161667

[B12] Chareyre S. Mandin P. (2018). Bacterial iron homeostasis regulation by sRNAs. *Microbiol. Spectr.* 6 1–15. 10.1128/microbiolspec.RWR-0010-2017 29573257 PMC11633579

[B13] Chen J. Morita T. Gottesman S. (2019). Regulation of transcription termination of small RNAs and by small RNAs: molecular mechanisms and biological functions. *Front. Cell. Infect. Microbiol.* 9:201. 10.3389/fcimb.2019.00201 31249814 PMC6582626

[B14] Conway T. Cohen P. S. (2015). Commensal and pathogenic *Escherichia coli* metabolism in the gut. *Microbiol Spectr.* 3:10.1128/microbiolspec.MBP-0006-2014. 10.1128/9781555818883.ch16 PMC4510460 26185077

[B15] Corcoran C. P. Podkaminski D. Papenfort K. Urban J. H. Hinton J. C. D. Vogel J. (2012). Superfolder GFP reporters validate diverse new mRNA targets of the classic porin regulator, MicF RNA. *Mol. Microbiol.* 84 428–445. 10.1111/j.1365-2958.2012.08031.x 22458297

[B16] Cornelissen C. N. (2018). Subversion of nutritional immunity by the pathogenic *Neisseriae*. *Pathog. Dis.* 76:ftx112. 10.1093/femspd/ftx112 29045638 PMC6251569

[B17] Curtis M. M. Hu Z. Klimko C. Narayanan S. Deberardinis R. Sperandio V. (2014). The gut commensal *Bacteroides thetaiotaomicron* exacerbates enteric infection through modification of the metabolic landscape. *Cell Host Microbe* 16 759–769. 10.1016/j.chom.2014.11.005 25498343 PMC4269104

[B18] De Lay N. Gottesman S. (2009). The crp-activated small noncoding regulatory RNA CyaR (RyeE) links nutritional status to group behavior. *J. Bacteriol.* 191 461–476. 10.1128/JB.01157-08 18978044 PMC2620814

[B19] Evfratov S. A. Osterman I. A. Komarova E. S. Pogorelskaya A. M. Rubtsova M. P. Zatsepin T. S. (2017). Application of sorting and next generation sequencing to study 5’-UTR influence on translation efficiency in *Escherichia coli*. *Nucleic Acids Res.* 45 3487–3502. 10.1093/nar/gkw1141 27899632 PMC5389652

[B20] Evstatiev R. Gasche C. (2012). Iron sensing and signalling. *Gut* 61 933–952. 10.1136/gut.2010.214312 22016365

[B21] Fischbach M. A. Sonnenburg J. L. (2011). Eating for two: how metabolism establishes interspecies interactions in the gut. *Cell Host Microbe* 10 336–347. 10.1016/j.chom.2011.10.002 22018234 PMC3225337

[B22] Förstner K. U. Vogel J. Sharma C. M. (2014). READemption-a tool for the computational analysis of deep-sequencing-based transcriptome data. *Bioinformatics* 30 3421–3423. 10.1093/bioinformatics/btu533 25123900

[B23] Fournier C. Smith A. Delepelaire P. (2011). Haem release from haemopexin by HxuA allows *Haemophilus influenzae* to escape host nutritional immunity. *Mol. Microbiol.* 80 133–148. 10.1111/j.1365-2958.2011.07562.x 21276097

[B24] Garcia E. C. Brumbaugh A. R. Mobley H. L. T. (2011). Redundancy and specificity of *Escherichia coli* iron acquisition systems during urinary tract infection. *Infect. Immun.* 79 1225–1235. 10.1128/iai.01222-10 21220482 PMC3067483

[B25] Gyles C. L. (2007). Shiga toxin-producing *Escherichia coli*: an overview. *J. Anim. Sci.* 85 E45–E62. 10.2527/jas.2006-508 17085726

[B26] Hagan E. C. Mobley H. L. T. (2009). Haem acquisition is facilitated by a novel receptor Hma and required by uropathogenic *Escherichia coli* for kidney infection. *Mol. Microbiol.* 71 79–91. 10.1111/j.1365-2958.2008.06509.x 19019144 PMC2736550

[B27] Hood M. I. Skaar E. P. (2012). Nutritional immunity: transition metals at the pathogen-host interface. *Nat. Rev. Microbiol.* 10 525–537. 10.1038/nrmicro2836 22796883 PMC3875331

[B28] Kaper J. B. Nataro J. P. Mobley H. L. (2004). Pathogenic *Escherichia coli*. *Nat. Rev. Microbiol.* 2 123–140. 10.1038/nrmicro818 15040260

[B29] Karpman D. Ståhl A.-L. (2014). Enterohemorrhagic *Escherichia coli* pathogenesis and the host response. *Microbiol. Spectr.* 2 1–15. 10.1128/microbiolspec.EHEC-0009-2013 26104359

[B30] Kim D. Hong J. S. J. Qiu Y. Nagarajan H. Seo J. H. Cho B. K. (2012). Comparative analysis of regulatory elements between *Escherichia coli* and *Klebsiella pneumoniae* by genome-wide transcription start site profiling. *PLoS Genet.* 8:e1002867. 10.1371/journal.pgen.1002867 22912590 PMC3415461

[B31] Kouse A. B. Righetti F. Kortmann J. Narberhaus F. Murphy E. R. (2013). RNA-mediated thermoregulation of iron-acquisition genes in *Shigella dysenteriae* and pathogenic *Escherichia coli*. *PLoS One* 8:e63781. 10.1371/journal.pone.0063781 23704938 PMC3660397

[B32] LaMattina J. W. Delrossi M. Uy K. G. Keul N. D. Nix D. B. Neelam A. R. (2017). Anaerobic heme degradation: ChuY is an anaerobilin reductase that exhibits kinetic cooperativity. *Biochemistry* 56 845–855. 10.1021/acs.biochem.6b01099 28045510 PMC7210504

[B33] LaMattina J. W. Nix D. B. Lanzilotta W. N. (2016). Radical new paradigm for heme degradation in *Escherichia coli* O157:H7. *Proc. Natl. Acad. Sci. U.S.A.* 113 12138–12143. 10.1073/pnas.1603209113 27791000 PMC5087033

[B34] Li H. Durbin R. (2010). Fast and accurate long-read alignment with Burrows-Wheeler transform. *Bioinformatics* 26 589–595. 10.1093/bioinformatics/btp698 20080505 PMC2828108

[B35] Li H. Handsaker B. Wysoker A. Fennell T. Ruan J. Homer N. (2009). The Sequence Alignment/Map format and SAMtools. *Bioinformatics* 25 2078–2079. 10.1093/bioinformatics/btp352 19505943 PMC2723002

[B36] Li L. Huang D. Cheung M. K. Nong W. Huang Q. Kwan H. S. (2013). BSRD: a repository for bacterial small regulatory RNA. *Nucleic Acids Res.* 41 233–238. 10.1093/nar/gks1264 23203879 PMC3531160

[B37] Lorenz R. Bernhart S. H. Höner zu Siederdissen C. Tafer H. Flamm C. Stadler P. F. (2011). ViennaRNA Package 2.0. *Algorithms Mol. Biol.* 6 122–128. 10.1186/1748-7188-6-26 22115189 PMC3319429

[B38] Mann M. Wright P. R. Backofen R. (2017). IntaRNA 2.0: enhanced and customizable prediction of RNA-RNA interactions. *Nucleic Acids Res.* 45 W435–W439. 10.1093/nar/gkx279 28472523 PMC5570192

[B39] Massé E. Gottesman S. (2002). A small RNA regulates the expression of genes involved in iron metabolism in *Escherichia coli*. *Proc. Natl. Acad. Sci. U.S.A.* 99 4620–4625. 10.1073/pnas.032066599 11917098 PMC123697

[B40] Massé E. Salvail H. Desnoyers G. Arguin M. (2007). Small RNAs controlling iron metabolism. *Curr. Opin. Microbiol.* 10 140–145. 10.1016/j.mib.2007.03.013 17383226

[B41] Miranda R. L. Conway T. Leatham M. P. Chang D. E. Norris W. E. Allen J. H. (2004). Glycolytic and gluconeogenic growth of *Escherichia coli* O157:H7 (EDL933) and *E. coli* K-12 (MG1655) in the mouse intestine. *Infect. Immun.* 72 1666–1676. 10.1128/IAI.72.3.1666-1676.2004 14977974 PMC355998

[B42] Moon K. Gottesman S. (2011). Competition among Hfq-binding small RNAs in *Escherichia coli*. *Mol. Microbiol.* 82 1545–1562. 10.1111/j.1365-2958.2011.07907.x 22040174 PMC7394283

[B43] Morgenthau A. Pogoutse A. Adamiak P. Moraes T. F. Schryvers A. B. (2013). Bacterial receptors for host transferrin and lactoferrin: molecular mechanisms and role in host-microbe interactions. *Future Microbiol.* 8 1575–1585. 10.2217/fmb.13.125 24266357

[B44] Nagy G. Dobrindt U. Kupfer M. Emody L. Karch H. Hacker J. (2001). Expression of hemin receptor molecule *chuA* is influenced by rfaH in uropathogenic *E. coli* strain 356. *Infect. Immun.* 69 1924–1928. 10.1128/IAI.69.3.1924 11179376 PMC98105

[B45] Naville M. Ghuillot-Gaudeffroy A. Marchais A. Gautheret D. (2011). ARNold: a web tool for the prediction of rho-independent transcription terminators. *RNA Biol.* 8 10–13. 10.4161/rna.8.1.13346 21282983

[B46] Njoroge J. W. Nguyen Y. Curtis M. M. Moreira C. G. Sperandio V. (2012). Virulence meets metabolism: Cra and KdpE gene regulation in enterohemorrhagic *Escherichia coli*. *mBio* 3:e00280-12. 10.1128/mBio.00280-12 23073764 PMC3482499

[B47] Oglesby-Sherrouse A. G. Murphy E. R. (2013). Iron-responsive bacterial small RNAs: variations on a theme. *Metallomics* 5 276–286. 10.1039/c3mt20224k 23340911 PMC3612141

[B48] Ong S. T. Shan Ho J. Z. Ho B. Ding J. L. (2006). Iron-withholding strategy in innate immunity. *Immunobiology* 211 295–314. 10.1016/j.imbio.2006.02.004 16697921

[B49] Peters J. M. Mooney R. A. Grass J. A. Jessen, Tran F. Landick R. (2012). Rho and NusG suppress pervasive antisense transcription in *E. coli*. *Genes Dev.* 26 2621–2633. 10.1101/gad.196741.112.The 23207917 PMC3521622

[B50] Peters J. M. Mooney R. A. Kuan P. F. Rowland J. L. Keles S. Landick R. (2009). Rho directs widespread termination of intragenic and stable RNA transcription. *Proc. Natl. Acad. Sci. U.S.A.* 106 15406–15411. 10.1073/pnas.0903846106 19706412 PMC2741264

[B51] Quinlan A. R. Hall I. M. (2010). BEDTools: a flexible suite of utilities for comparing genomic features. *Bioinformatics* 26 841–842. 10.1093/bioinformatics/btq033 20110278 PMC2832824

[B52] Reuter J. S. Mathews D. H. (2010). RNAstructure: software for RNA secondary structure prediction and analysis. *BMC Bioinformatics* 11:129. 10.1186/1471-2105-11-129 20230624 PMC2984261

[B53] Ridley K. A. Rock J. D. Li Y. Ketley J. M. (2006). Heme utilization in *Campylobacter jejuni*. *J. Bacteriol.* 188 7862–7875. 10.1128/JB.00994-06 16980451 PMC1636299

[B54] Righetti F. Nuss A. M. Twittenhoff C. Beele S. Urban K. Will S. (2016). Temperature-responsive in vitro RNA structurome of *Yersinia pseudotuberculosis*. *Proc. Natl. Acad. Sci. U.S.A.* 113 7237–7242. 10.1073/pnas.1523004113 27298343 PMC4932938

[B55] Rossi M. S. Fetherston J. D. Létoffé S. Carniel E. Perry R. D. Ghigo J. M. (2001). Identification and characterization of the hemophore-dependent heme acquisition system of *Yersinia pestis*. *Infect. Immun.* 69 6707–6717. 10.1128/IAI.69.11.6707-6717.2001 11598042 PMC100047

[B56] Ryu S. Ramseier T. M. Michotey V. Saier M. H. Garges S. (1995). Effect of the FruR regulator on transcription of the *pts* operon in *Escherichia coli*. *J. Biol. Chem.* 270 2489–2496. 10.1074/jbc.270.6.2489 7852310

[B57] Santiago-Frangos A. Kavita K. Schu D. J. Gottesman S. Woodson S. A. (2016). C-terminal domain of the RNA chaperone Hfq drives sRNA competition and release of target RNA. *Proc. Natl. Acad. Sci. U.S.A.* 113 E6089–E6096. 10.1073/pnas.1613053113 27681631 PMC5068269

[B58] Schu D. J. Zhang A. Gottesman S. Storz G. (2015). Alternative Hfq-sRNA interaction modes dictate alternative mRNA recognition. *EMBO J.* 34 2557–2573. 10.15252/embj.201591569 26373314 PMC4609186

[B59] Sedlyarova N. Shamovsky I. Bharati B. K. Epshtein V. Chen J. Gottesman S. (2016). sRNA-mediated control of transcription termination in *E. coli*. *Cell* 167 111–121.e13. 10.1016/j.cell.2016.09.004 27662085 PMC5040353

[B60] Segura A. Bertin Y. Durand A. Benbakkar M. Forano E. (2021). Transcriptional analysis reveals specific niche factors and response to environmental stresses of enterohemorrhagic *Escherichia coli* O157:H7 in bovine digestive contents. *BMC Microbiol.* 21:284. 10.1186/s12866-021-02343-7 34663220 PMC8524897

[B61] Segura A. Bertoni M. Auffret P. Klopp C. Bouchez O. Genthon C. (2018). Transcriptomic analysis reveals specific metabolic pathways of enterohemorrhagic *Escherichia coli* O157:H7 in bovine digestive contents. *BMC Genomics* 19:766. 10.1186/s12864-018-5167-y 30352567 PMC6199705

[B62] Sharma C. M. Hoffmann S. Darfeuille F. Reignier J. Findeiß S. Sittka A. (2010). The primary transcriptome of the major human pathogen *Helicobacter pylori*. *Nature* 464 250–255. 10.1038/nature08756 20164839

[B63] Silva I. J. Barahona S. Eyraud A. Lalaouna D. Figueroa-Bossi N. Massé E. (2019). SraL sRNA interaction regulates the terminator by preventing premature transcription termination of rho mRNA. *Proc. Natl. Acad. Sci. U.S.A.* 116 3042–3051. 10.1073/pnas.1811589116 30718400 PMC6386699

[B64] Skaar E. P. (2010). The battle for iron between bacterial pathogens and their vertebrate hosts. *PLoS Pathog.* 6:e1000949. 10.1371/journal.ppat.1000949 20711357 PMC2920840

[B65] Snider T. A. Fabich A. J. Conway T. Clinkenbeard K. D. (2009). *E. coli* O157:H7 catabolism of intestinal mucin-derived carbohydrates and colonization. *Vet. Microbiol.* 136 150–154. 10.1016/j.vetmic.2008.10.033 19095384

[B66] Soper T. J. Woodson S. (2008). The rpoS mRNA leader recruits Hfq to facilitate annealing with DsrA sRNA. *RNA* 14 1907–1917. 10.1261/rna.1110608 18658123 PMC2525945

[B67] Sy B. M. Lan R. Tree J. J. (2020). Early termination of the Shiga toxin transcript generates a regulatory small RNA. *Proc. Natl. Acad. Sci. U.S.A.* 117 25055–25065. 10.1073/pnas.2006730117 32968018 PMC7547250

[B68] Tollervey D. Mattaj I. W. (1987). Fungal small nuclear ribonucleoproteins share properties with plant and vertebrate U-snRNPs. *EMBO J.* 6 469–476. 10.1002/j.1460-2075.1987.tb04777.x 2953599 PMC553418

[B69] Torres A. G. Payne S. M. (1997). Haem iron-transport system in enterohaemorrhagic *Escherichia coli* O157:H7. *Mol. Microbiol.* 23 825–833. 10.1046/j.1365-2958.1997.2641628.x 9157252

[B70] Torres A. G. Redford P. Welch R. A. Payne S. M. (2001). TonB-dependent systems of uropathogenic *Escherichia coli*: Aerobactin and heme transport and TonB are required for virulence in the mouse. *Infect. Immun.* 69 6179–6185. 10.1128/IAI.69.10.6179-6185.2001 11553558 PMC98749

[B71] Tree J. J. Granneman S. McAteer S. P. Tollervey D. Gally D. L. (2014). Identification of bacteriophage-encoded anti-sRNAs in pathogenic *Escherichia coli*. *Mol. Cell* 55 199–213. 10.1016/j.molcel.2014.05.006 24910100 PMC4104026

[B72] Urban J. H. Vogel J. (2007). Translational control and target recognition by *Escherichia coli* small RNAs *in vivo*. *Nucleic Acids Res.* 35 1018–1037. 10.1093/nar/gkl1040 17264113 PMC1807950

[B73] Wagner E. G. H. Romby P. (2015). “Small RNAs in bacteria and archaea: Who they are, what they do, and how they do it,” in *Advances in Genetics*, eds Friedmann T. Dunlap J. C. Goodwin S. F. (Waltham, MA: Academic Press). 10.1016/bs.adgen.2015.05.001 26296935

[B74] Waldminghaus T. Heidrich N. Brantl S. Narberhaus F. (2007). FourU: a novel type of RNA thermometer in *Salmonella*. *Mol. Microbiol.* 65 413–424. 10.1111/j.1365-2958.2007.05794.x 17630972

[B75] Wang D. McAteer S. P. Wawszczyk A. B. Russell C. D. Tahoun A. Elmi A. (2018). An RNA-dependent mechanism for transient expression of bacterial translocation filaments. *Nucleic Acids Res.* 46 3366–3381. 10.1093/nar/gky096 29432565 PMC5909449

[B76] Waters L. S. Storz G. (2009). Regulatory RNAs in bacteria. *Cell* 136 615–628. 10.1016/j.cell.2009.01.043 19239884 PMC3132550

[B77] Waters S. A. McAteer S. P. Kudla G. Pang I. Deshpande N. P. Amos T. G. (2017). Small RNA interactome of pathogenic *E. coli* revealed through crosslinking of RNase E. *EMBO J.* 36 374–387. 10.15252/embj.201694639 27836995 PMC5286369

[B78] Weber G. G. Kortmann J. Narberhaus F. Klose K. E. (2014). RNA thermometer controls temperature-dependent virulence factor expression in *Vibrio cholerae*. *Proc. Natl. Acad. Sci. U.S.A.* 111 14241–14246. 10.1073/pnas.1411570111 25228776 PMC4191814

[B79] Weinberg E. D. (1975). Nutritional Immunity: host’s attempt to withhold iron from microbial invaders. *J. Am. Med. Assoc.* 231 39–41. 10.1093/ajcn/30.9.1485 1243565

[B80] Wright P. R. Georg J. Mann M. Sorescu D. A. Richter A. S. Lott S. (2014). CopraRNA and IntaRNA: predicting small RNA targets, networks and interaction domains. *Nucleic Acids Res.* 42 119–123. 10.1093/nar/gku359 24838564 PMC4086077

[B81] Wyckoff E. E. Duncan D. Torres A. G. Mills M. Maase K. Payne S. M. (1998). Structure of the *Shigella dysenteriae* haem transport locus and its phylogenetic distribution in enteric bacteria. *Mol. Microbiol.* 28 1139–1152. 10.1046/j.1365-2958.1998.00873.x 9680204

[B82] Xu J. Bjursell M. K. Himron J. Deng S. Carmichael L. K. Chaing H. C. (2003). A genomic view of the human-*Bacteroides thetaiotaomicron* symbiosis. *Science* 299 2074–2076. 10.1126/science.1080029 12663928

[B83] Zambolin S. Clantin B. Chami M. Hoos S. Haouz A. Villeret V. (2016). Structural basis for haem piracy from host haemopexin by *Haemophilus influenzae*. *Nat. Commun.* 7:11590. 10.1038/ncomms11590 27188378 PMC4873976

[B84] Zhang A. Schu D. J. Tjaden B. C. Storz G. Gottesman S. (2013). Mutations in interaction surfaces differentially impact *E. coli* Hfq association with small RNAs and their mRNA targets. *J. Mol. Biol.* 425 3678–3697. 10.1016/j.jmb.2013.01.006 23318956 PMC3640674

[B85] Zhang Z. Aboulwafa M. Saier M. H. (2014). Regulation of crp gene expression by the catabolite repressor/activator, Cra, in *Escherichia coli*. *J. Mol. Microbiol. Biotechnol.* 24 135–141. 10.1159/000362722 24923415 PMC4125524

[B86] Zuker M. (2003). Mfold web server for nucleic acid folding and hybridization prediction. *Nucleic Acids Res.* 31 3406–3415. 10.1093/nar/gkg595 12824337 PMC169194

[B87] Zwiefka A. Kohn H. Widger W. R. (1993). Transcription termination factor rho: the site of bicyclomycin inhibition in *Escherichia coli*. *Biochemistry* 32 3564–3570. 10.1021/bi00065a007 8466900

