# Supplementary figures and images for: The Small RNA CyaR Activates Translation of the Outer Membrane Haem Receptor chuA in Enterohemorrhagic Escherichia coli
Source: Front Microbiol. 2022 Mar 29;13:821196. doi: 10.3389/fmicb.2022.821196 (PMC9002310; doi:10.3389/fmicb.2022.821196)

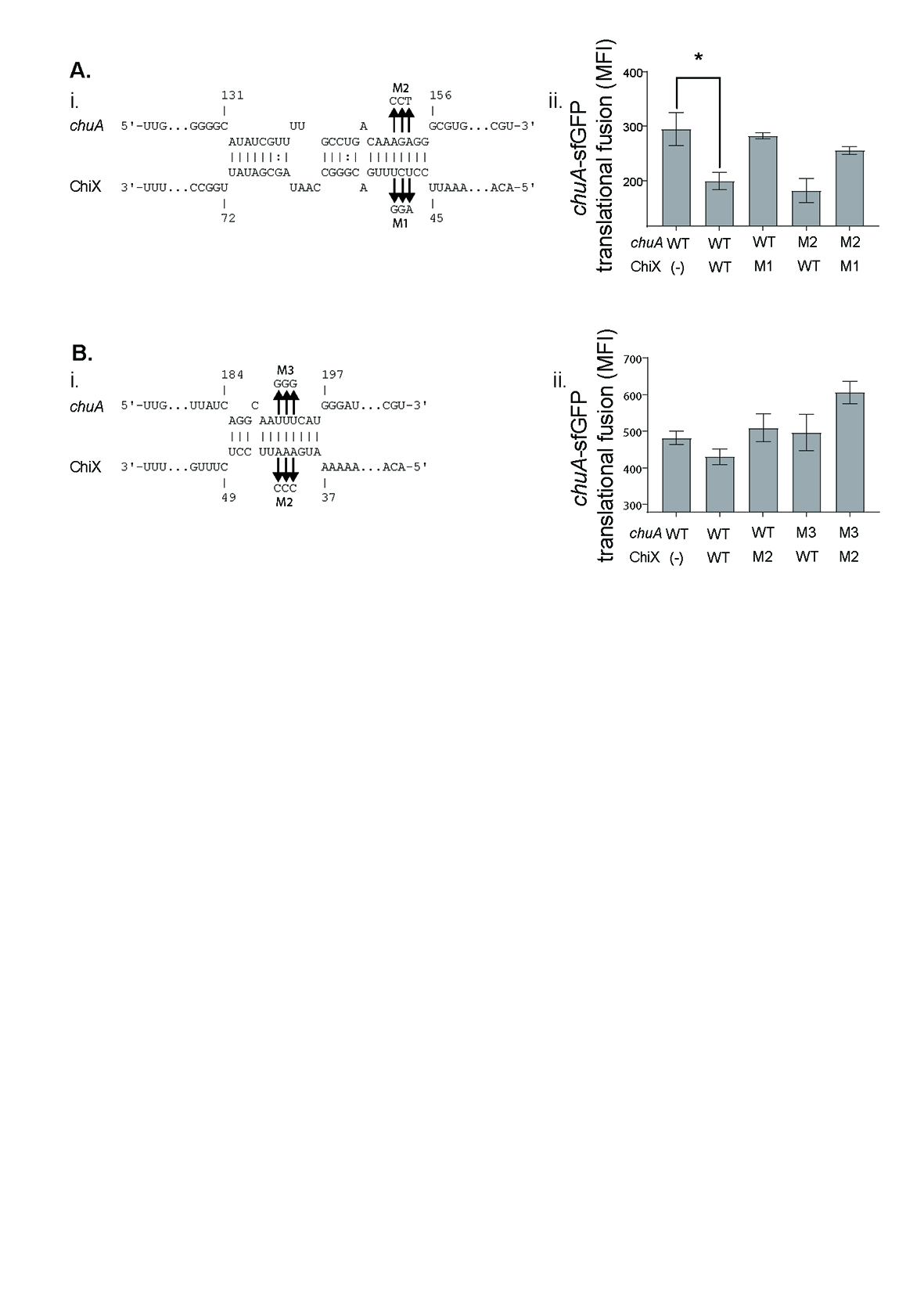

Supplement: Supplementary Figure 1 — ChiX indirectly represses chuA translation. (A,B) (Left) IntaRNA prediction of the chuA–ChiX interaction. Compensatory point mutations predicted to disrupt the interaction are indicated by the arrows. (Right) Fluorescence measurements of wild-type or mutant chuA-sfGFP translational fusions in the presence and absence of wild-type or mutant ChiX overexpression plasmid. Measurements are the mean median fluorescence intensity of three biological replicates. [file Image_1.TIFF]

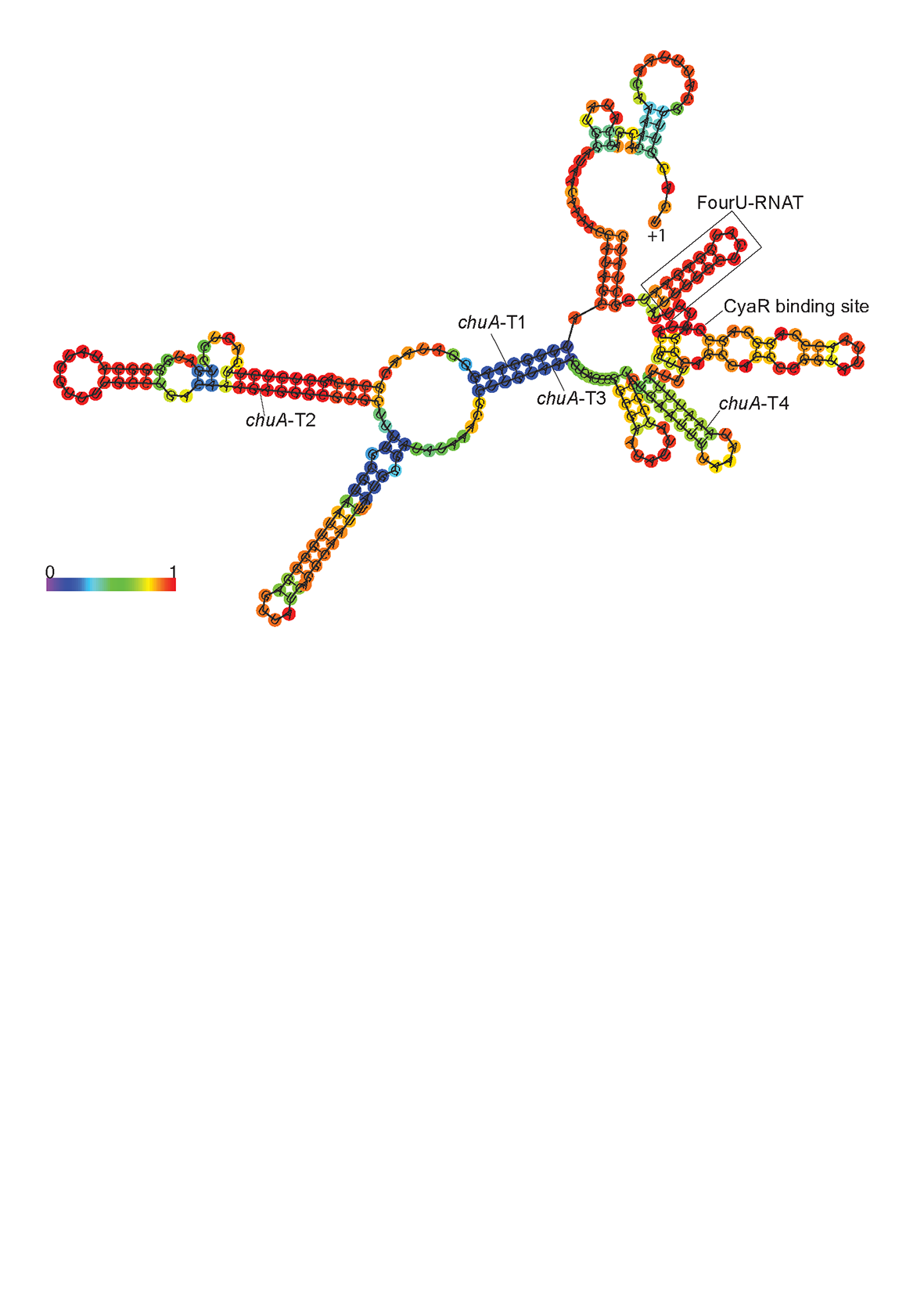

Supplement: Supplementary Figure 2 — Secondary structure of the chuA 5′UTR as predicted by RNAfold. The +1 site, CyaR binding site, FourU RNA thermometer and sites where truncations were made are indicated. [file Image_2.TIFF]
